# 自体造血干细胞移植治疗M蛋白相关性杆状体肌病1例

**DOI:** 10.3760/cma.j.issn.0253-2727.2022.01.016

**Published:** 2022-01

**Authors:** 文灏 董, 召 李, 冰 赵, 成录 袁

**Affiliations:** 1 山东大学齐鲁医院（青岛）血液科，青岛 266035 Department of Hematology, Qilu Hospital (Qingdao), Cheeloo College of Medicine, Shandong University, Qingdao 266035, China; 2 山东大学齐鲁医院（青岛）神经内科，青岛 266035 Department of Neurology, Qilu Hospital (Qingdao), Cheeloo College of Medicine, Shandong University, Qingdao 266035, China

患者，男，63岁，1年前开始出现左下肢无力、变细，继而右下肢也出现同样症状，随后双上肢抬举费力，右上肢近端肌肉萎缩，入院前出现吞咽困难。既往腰椎间盘突出5年、颈椎间盘突出1年，家族史阴性。入院查体：行走呈鸭步。双上肢肌力：肩外展：左侧3级，右侧2级；屈肘：左侧4+级，右侧4-级；伸肘：左侧4级，右侧3级；余正常。双下肢肌力：屈髋：左侧3级，右侧3级；屈膝：左侧4级，右侧5级；伸膝：左侧4+级，右侧4+级；余正常。右上肢近端肌肉及左下肢肌肉明显萎缩，双侧翼状肩。全身肌肉无压痛。病理征（−）。心肌酶谱：肌酸激酶（CK）137 U/L（参考值38～174 U/L），乳酸脱氢酶（LDH）258 U/L（参考值91～245 U/L）；甲状腺功能六项正常；风湿系列正常；血生化：球蛋白43.7 g/L（参考值20～40 g/L）；血清免疫固定电泳游离L轻链阳性；尿免疫固定电泳阴性；血清蛋白电泳阴性；心脏超声、脑动脉超声、颈部超声均未见异常；胸部CT：双肺条索影；磁共振成像（MRI）：双侧股前肌群、股内侧肌群、股后肌群对称性略萎缩，双侧小腿周围肌肉，包括外侧肌群及后部肌群对称性略萎缩，双侧盆腔周围臀区肌群、髂区肌群及股前肌群对称性轻度萎缩。骨髓象：浆细胞占1％，形态大致正常。骨髓流式细胞术未见异常浆细胞。肌电图：双侧股四头肌、肱二头肌、左侧三角肌静息下可见不同程度正尖样波或纤颤样波，部分肌肉可见窄小电位、左侧腓肠肌可见少量宽大电位，部分所检肌呈肌源性损害+自发电位表现。

左股四头肌活检病理：HE染色（图1A）：肌纤维大小明显不等，小纤维多呈长条形，小多边形及小角形；偶见坏死及再生肌纤维，未见肥大劈裂及高收缩肌纤维；可见散在及成小群分布的萎缩肌纤维；部分肌纤维可见空泡及嗜碱性物质沉积；内核纤维未见明显增多，未见核袋；肌纤维内膜轻度增生，肌间质未见灶性炎性细胞浸润。还原型辅酶-1染色（图1B）：较多肌纤维可见分叶肌纤维，可见肌浆块。改良Gomori染色（图1C）：可见较多肌纤维内嗜碱性物质红染，部分肌纤维内可见杆状体，以萎缩肌纤维为主。环氧酶（COX）染色：偶见COX酶活性缺失肌纤维。琥珀酸脱氢酶/细胞色素氧化酶双染色：可见4条蓝纤维。三磷酸腺苷酶染色：两型纤维均有萎缩，Ⅱ型纤维优势。刚果红染色阴性（图1D）。结论：符合肌源性损害病理改变，结合临床考虑成人杆状体肌病可能。最终诊断：M蛋白相关性成人晚发型杆状体肌病。

**图1:**
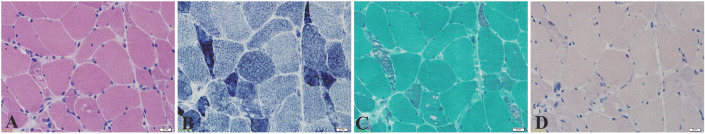
M蛋白相关性杆状体肌病患者肌肉活检病理（400×） A：HE染色见肌纤维明显大小不等，散在及成小群分布的萎缩肌纤维，部分肌纤维可见空泡及嗜碱性物质沉积；B：还原型辅酶-1染色见较多肌纤维可见分叶肌纤维，可见肌浆块；C：改良Gomori染色见较多肌纤维内嗜碱性物质红染，部分肌纤维内可见杆状体，以萎缩肌纤维为主；D：刚果红染色未见异常

自体造血干细胞移植治疗过程：以大剂量美法仑（140 mg/m^2^，−2 d）进行预处理。回输单个核细胞23×10^8^/kg、CD34^+^细胞2.7×10^6^/kg。移植后11 d造血重建，顺利出仓。移植后1个月，复查血清免疫固定电泳阴性，肌力好转不明显；移植后5个月，血清免疫固定电泳阴性，双上肢力量较前明显好转。

讨论：本例患者是国内首次接受自体造血干细胞移植治疗的M蛋白相关性杆状体肌病病例。患者移植后1个月复查血液学指标转为阴性，移植后5个月肌肉力量尤其是双上肢肌力较移植前明显改善，证实自体造血干细胞移植为该病安全、有效的治疗方法。

